# Pathogenic Potential and Antimicrobial Resistance Profile of *Staphylococcus aureus* in Milk and Beef from the Northwest and Southwest Regions of Cameroon

**DOI:** 10.1155/2020/6015283

**Published:** 2020-10-27

**Authors:** Marie Ebob Agbortabot Bissong, Brandon Fonyuy Tahnteng, Collins Njie Ateba, Jane-Francis Tatah Kihla Akoachere

**Affiliations:** ^1^Department of Biomedical Science, University of Bamenda, P.O. Box 39, Bambili, Cameroon; ^2^Department of Microbiology and Parasitology, University of Buea, P.O. Box 63, Buea, Cameroon; ^3^Food Security and Safety Niche Area, North-West University, Mafikeng Campus, Private Bag X2046, Mmabatho 2735, South Africa

## Abstract

*Staphylococcus aureus* is a major foodborne pathogen and commensal of the skin and mucous membranes of animals and humans. Its virulence relies on the production of a variety of toxins resistant to denaturing conditions. Increasing reports of *S. aureus* food poisoning and contamination of foods of animal origin elsewhere necessitates the investigation of these foods in Cameroon, to implement safety measures. This cross-sectional study evaluated *S. aureus* contamination in milk and beef in the Northwest and Southwest Regions of Cameroon, where cow milk is usually not pasteurized before consumption, and beef is the main source of protein. The distribution of antibiotic-resistant isolates and those with enterotoxin-producing potential was also investigated to provide data of public health and food safety benefit. *S. aureus* was isolated from 39 raw milk and 250 beef samples by standard methods. Confirmation of isolates was by PCR to detect the *nuc* gene. *S. aureus* was investigated for classical staphylococcal enterotoxin (SE) genes (*sea*, *seb*, *sec*, *sed*, and *see*) by PCR. Their susceptibility to 9 antibiotics was tested by the disk diffusion method. The chi-square test was used to compare the contamination of samples, antibiotic resistance, and the distribution of SE genes. *S. aureus* was isolated from 11.1% of samples. Contamination was higher in milk (48%) than in beef (5.2%) (*P* < 0.001). The *sea* was the most frequently (90%) harboured gene. A large proportion of isolates (88%) harboured more than one virulence gene. Isolates were generally resistant to erythromycin (82%), vancomycin (80%), tetracycline (76%), and oxacillin (74%). Multidrug resistance (MDR) was common (92%). Milk and beef samples in study area were contaminated with MDR enterotoxigenic *S. aureus* strains and may constitute a potential hazard to consumers. Thus, the need for implementation of proper hygienic measures when handling these products and pasteurization of milk cannot be overemphasized.

## 1. Introduction

The growing population of Cameroon has prompted a corresponding increase in the demand for food in general, including milk and meat. Unfortunately, food particularly that from animal origin could be contaminated with pathogenic microbes, leading to severe health consequences [1]. The high nutritional composition and neutral pH of milk and beef make them an excellent growth media for microbes, hence vehicles for transmission of foodborne pathogens to humans [2].

*S. aureus*, a Gram-positive skin and mucous membrane commensal of humans and animals [3, 4], is highly incriminated in cases of food poisoning associated with the consumption of contaminated milk and other animal products [5]. Recent reports of staphylococcal food poisoning (SFP) worldwide [6–9] have raised public health concerns regarding the contamination of food by this organism. *S. aureus* contamination of food including raw milk and beef generally results from infected cattle, infected food handlers, and contaminated utensils or environment [7]. Consumption of such contaminated foods may result in staphylococcal food poisoning. Consequently, the safety of these basic food products that are consumed daily needs to be investigated.

Staphylococcal food poisoning results from the consumption of food containing minute quantities of one or more preformed staphylococcal enterotoxins (SEs). There are more than 20 distinct SEs; however, only a few of them (the classical enterotoxins (SEA, SEB, SEC, SED, and SEE)) have been studied in depth [10]. Among these, SEA and SED have been reported to be the most common toxins in staphylococcus-related food poisoning worldwide [8]. New enterotoxins with emetic activity enterotoxin-like types that lack emetic activity have been described in *S. aureus* [11]. While the role of the nonclassical SEs is not fully elucidated, SEF has been implicated in toxic shock syndrome [12]. SEH has also been identified as one of the main causes of massive food poisoning associated with the consumption of reconstituted milk [13]. The pathogenicity of *S. aureus* harbouring SEs is enhanced by the fact that these enterotoxins are resistant to denaturing conditions such as low pH, low temperature, heating, and digestion by proteolytic enzymes which allows them to remain intact in food, contributing to the high prevalence of SFP outbreaks [14].

Most genes coding for SEs are located on mobile genetic elements such as plasmids (SED), prophage (SEA and SEE), chromosome (SEB), or pathogenicity islands on the chromosome (SEB and SEC) [14, 15]. Consequently, horizontal transfer between strains can occur, modifying the ability of *S. aureus* strains to cause disease and contributing to pathogen evolution [14]. SEs are produced during all phases of growth (SEA and SED) or only as secondary metabolites in late exponential or in stationary phase (SEB and SEC). Most strains of *S. aureus* produce one or more enterotoxins; most of which are superantigens, stimulating large populations of T cells that result in acute toxic shock [16].

Aside the severe consequences of SEs, a more disturbing concern is the emergence of multidrug-resistant strains of *S. aureus* [17]. The use and misuse of antibiotics for therapeutic and prophylaxis purposes in farms and as growth promoters in animal feed may drive the selection of antibiotic-resistant strains of *S. aureus* that eventually end up in foods of animal origin [18, 19]. The use of antibiotics in animal husbandry is strongly discouraged due to its long-term effect resulting from the development of resistance. To this effect, some countries worldwide have placed a band on the use of antibiotics as growth promoters in animal feed as is the case of the EU. However, in other areas especially in the developing countries, antibiotics are incorporated either in feed or water in order to reduce mortality in farms. In Cameroon, in Dec 2018, the Minister of Livestock and Animal Husbandry made a statement denouncing the use of antibiotics in animal feed [20]. So far, there is still no legislature to this effect and no monitoring system exists to regulate the use of antibiotics in animal husbandry in Cameroon. The fact is that antimicrobial agents are readily available in local drug stores without prescription and the absence of a structured program to control antimicrobial usage makes it possible for these agents to be misused. Previous reports confirm the indiscriminate use of antibiotics in the agropastoral sector in Cameroon with corresponding high levels of antimicrobial residues in animals or animal products [21, 22]. Therefore, there is a need for a surveillance system to be instituted that will regulate the use of antibiotic in this setup.

*S. aureus* is notorious for its ability to develop resistance to multiple antibiotics, through a wide array of mechanisms. Of global concern is methicillin-resistant *S. aureus* (MRSA), strains of *S. aureus* which have acquired the *mecA* gene located on the mobile element of the staphylococcal chromosome cassette mec (SCC*mec*). The *mecA* gene encodes for the penicillin-binding protein 2a (PBP2a) which confers resistance to multiple antibiotics particularly *β*-lactam antibiotics [23].

Several studies have shown foods of animal origin to be an important reservoir of *S. aureus* [24–28], and there are increasing reports on foodborne disease outbreaks linked to *S. aureus.* In Cameroon, most reports on *S. aureus* are mainly from clinical samples and hospital environment [29–32]. Data on this pathogen as a food safety concern in Cameroon are therefore insufficient. A few studies have investigated the microbiological quality of meat and isolated *S. aureus* as a contaminant [33, 34]. Afnabi et al. [35] studied the antimicrobial susceptibility of coagulase-positive *S. aureus* isolates from meat in northern Cameroon. These studies did not investigate the virulence potential of the organism. Fonou et al. [46] carried out whole genome analysis to describe the genetic environment and genetic lineages of MRSA in pigs in Cameroon. Also, data on contamination of milk is scarce. There is therefore a need for more studies aimed to investigate this organism as a food safety concern in Cameroon particularly as food safety critically affects health and hence the attainment of some of the Sustainable Development Goals (SDGs). The purpose of this study was to detect enterotoxin genes and assess the antibiotic resistance profiles of *S. aureus* from raw milk and beef, with the ultimate goal to ascertain the possible risks associated with the consumption of these products. Data from this study will be of public health relevance and might provide the basis for risk assessment as well as food safety.

## 2. Methods

### 2.1. Study Design and Study Area

**Figure 1:**
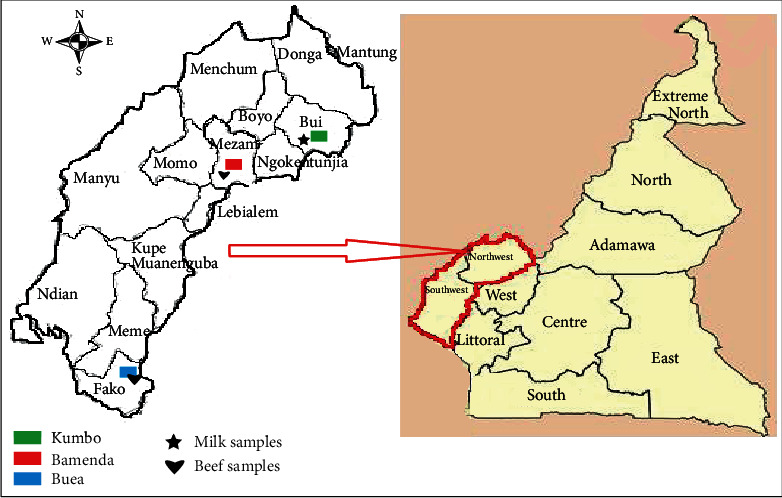
Regional and divisional maps of Cameroon showing the study location (adapted from Google Maps).

A cross-sectional study was used to determine the antibiotic resistance and enterotoxin-producing potentials of *S. aureus* isolated from raw milk from dairy farms in Kumbo and beef samples from abattoirs in Bamenda and Buea (Figure 1).

The study was conducted during 8-month periods, from April to November, 2018, in the Northwest Region (Bamenda and Kumbo) and Southwest Region (Buea), Cameroon. Both regions have two seasons: the rainy season which starts from April to October and the dry season, from November to March. The Northwest Region is found in the western highlands of Cameroon. It is a mountainous area with many plains and plateaus and grassland vegetation [36]. This geographical presentation and vegetation makes the area suitable for cattle grazing, and as a result, this region is known to be one of the major suppliers of beef to other parts of the country. Kumbo and Bamenda are the largest towns in the Northwest Region, with Bamenda being the regional capital. Kumbo is located about 2000 m above sea level and is 111 km away from Bamenda. The population of Kumbo is estimated to be over 53000 inhabitants while that of Bamenda over 393000 [37]. Cattle rearing is done mostly in the outskirts of these towns and in villages. Milk from dairy farms is sold in the Northwest Region and is consumed raw or processed to yoghourt or cheese. Beef is the main source of animal protein for individuals residing in these areas.

Buea is the capital city of the Southwest Region and the area is basically a forest zone; thus, cattle rearing is limited. Beef sold in Buea and other parts of the Southwest is obtained from cattle from the Northwest and the northern regions of the country. Buea is located on the eastern slopes of Mount Cameroon. The climate of Buea is humid with neighbourhoods at higher elevations having cooler temperatures, while the lower areas experience warmer temperatures. Extended periods of rainfall characterized by incessant drizzle which can at times last for weeks are common during the rainy season with damp fogs rolling off the mountain into the town.

### 2.2. Ethical Considerations

Ethical approval was obtained from the University of Buea Institutional Animal Care and Use Committee (Ref. No. 2017/02/UB/IACUC/BTU/FS). Verbal administrative authorizations were obtained from the Delegations of Livestock, Fisheries and Animal Industries for the Northwest and Southwest Regions. Approval for sample collection was received from dairy farm owners and managers of the abattoirs. The names of dairy farms and abattoirs were coded to ensure confidentiality.

### 2.3. Sampling and Sample Collection

The sample size for this study (approximately 300) was calculated using the formula described by Charan and Biswas [38] with an expected proportion of 32% (prevalence of isolation of *S. aureus* from beef) based on previous reports [39]. Selection of dairy farms was purposive in which two of the farms in Kumbo (S and T) with the highest number of lactating cattle were targeted and milk samples were collected from all the lactating cattle (40) in these farms.. About 50 mL of milk was collected aseptically by trained farm personnel from each lactating cow into sterile 50 mL collection tubes (Eppendorf, UK Ltd., Arlington, UK). Meanwhile, beef samples were collected from the major abattoirs in Bamenda (abattoir A) and Buea (abattoir B). Although cattle is being slaughtered on a daily basis in these abattoirs, these abattoirs have two specific days in a week in which the number of cattle slaughtered is highest. Hence, beef samples were collected twice weekly based on these days and selection of cattle/carcass was based on convenient sampling in which all (250) the cattle slaughtered were sampled. Approximately 10 grams of beef samples was collected aseptically into sterile plastic bags. The beef samples comprising lean meat were collected randomly in a pool from five different locations of each carcass. All samples were kept at 4°C and transported on ice to the laboratory for analysis.

The formula outlined below was used to determine the number of samples collected during the study [38]:

(1) $$\begin{array}{c} \text{Sample }\left(N\right)=\frac{{\left(Z_{1-\alpha /2}\right)}^{2}P\left(1-P\right)}{d^{2}}, \end{array}$$

where *Z*_1−*α*/2_ is the standard normal variate at 5% type I error (*P* < 0.05) and it is 0.05. *P* is the expected prevalence in population based on a previous study (32%). *d* is the absolute error or precision (which is 5%).

(2) $$\begin{array}{c} \text{Sample}\left(N\right)=\frac{{\left(1.96\right)}^{2}\left(0.32\right)\left(1–0.32\right)}{{\left(0.05\right)}^{2}}. \end{array}$$

Thus, the expected minimum number of samples to be collected was *N* = 334. From this, we estimated using approximately 340 samples.

### 2.4. Isolation and Identification of *S. aureus*

Five grams of each beef sample was macerated in a mortar and suspended in 45 mL peptone water (Liofilchem, Italy). One hundred microliters (100 *μ*L) was then spread-plated on mannitol salt agar (MSA) (Liofilchem, Italy), and plates were incubated at 37°C for 24 hours.

For milk samples, 100 *μ*L of each sample was added to 900 *μ*L of sterile peptone water and mixed. The tubes were incubated at 37°C overnight after which 100 *μ*L of culture was spread-plated unto MSA and incubated at 37°C for 24 hours. Plates were examined for characteristic yellow colonies. Pure isolates were characterized by gram staining, catalase, and coagulase tests as previously described [40] and preserved on nutrient agar slants at 4°C.

### 2.5. DNA Extraction

Genomic DNA of all presumptive isolates was extracted by a modified SDS-chloroform method as previously described [41]. Briefly, 5 mL of 24 h-old nutrient broth (Liofilchem, Italy) culture of isolates was centrifuged at 8000 rpm for 2 minutes and the supernatant was discarded. The resulting pellets were washed twice and resuspended with 400 *μ*L of 1 mM EDTA (pH, 8.0). Then, 1% lysozyme was added and the suspension was incubated at 37°C for 30 minutes. After, 400 *μ*L of lysis buffer (0.01 M Tris-HCL, 11.4 mM sodium citrate, 1 mM EDTA, and 1% sodium dodecyl sulfate) was added and vortexed vigorously for 1 minute. Then, 100 *μ*L of sodium acetate and 600 *μ*L of chloroform were added and tubes were centrifuged at 8000 rpm for 2 minutes. Four hundred microliters of the supernatant was later transferred to new sterile Eppendorf tubes, and 800 *μ*L of cold absolute ethanol (stored at -20°C) was added and mixed gently to precipitate the DNA. The precipitated DNA was then centrifuged at 13000 rpm for 1 minute, and the supernatant was discarded. The DNA tubes were later dried by inverting on sterile tissue paper. Fifty microliters of sterile TE buffer was added and vortexed to dissolve the DNA. Agarose gel electrophoresis was then used to confirm the presence of DNA. DNA was stored at -20°C for further analysis.

### 2.6. Confirmation of Isolates and Detection of Staphylococcal Enterotoxin Genes

To confirm isolates were *S. aureus*, PCR targeting the *nuc* gene was conducted using species-specific primers to the gene (Table 1) as described by Akindolire et al. [25]. Each reaction mixture of 25 *μ*L contained 12.5 *μ*L of PCR master mix, 11 *μ*L of PCR water, 1 *μ*L of genomic DNA, and 0.25 *μ*L of each oligonucleotide. Nuclease-free water was used as negative control. The PCR amplification protocol [21] included a total of 30 cycles ran under the following conditions: initial DNA denaturation at 94°C for 5 minutes, cycle DNA denaturation at 94°C for 30 seconds, annealing temperature 55°C for 30 seconds, and an extension at 72°C for 1 minute. A final extension at 72°C for 5 minutes and cooling to 4°C were performed.

PCR targeting classical SE genes (*sea*, *seb*, *sec*, *sed*, and *see*) was conducted using specific primers (Table 1). A reaction volume of 25 *μ*L containing 12.5 *μ*L of master mix, 11 *μ*L of nuclease-free water, 1 *μ*L of genomic DNA, and 0.25 *μ*L of the forward and reverse primers was used. All the reagents were purchased from Inqaba Biotec, Pretoria, South Africa. Amplification conditions were described by Akindolire et al. [25]. Two negative controls with PCR water were included in each PCR run. PCR products were resolved by electrophoresis on a 1% (*w*/*v*) agarose gel, visualized with a UV transilluminator, and photographed using a Gel Documentation-XR reader (Bio-Rad, Hercules, CA). A 100 bp DNA molecular weight marker (Fermentas, USA) was used to determine the amplicon size.

### 2.7. Antimicrobial Sensitivity Testing

Isolates were tested against a panel of nine (9) antibiotics belonging to seven different classes of antibiotics, using the Kirby-Bauer disk diffusion method as described by the Clinical and Laboratory Standards Institute [42]. Antimicrobial agents tested (Oxoid, Basingstoke, England) included tetracycline (30 *μ*g), neomycin (30 *μ*g), sulfamethoxazole (25 *μ*g), oxacillin (1 *μ*g), cefoxitin (30 *μ*g), ampicillin (10 *μ*g), amoxicillin (10 *μ*g), vancomycin (30 *μ*g), and erythromycin (15 *μ*g). Three to 4 pure colonies from an overnight nutrient agar culture were emulsified in 3 mL of normal saline and the turbidity adjusted to match that of a 0.5 McFarland standard. A sterile cotton wool swab was dipped into the standardized suspension of bacteria cells and used to evenly inoculate Mueller-Hinton agar (Liofilchem, Italy) plates. Plates were allowed to dry, and disks placed at least 15 mm apart and from the edge of the plate to prevent the overlapping of zones of inhibition. Plates were incubated for 24 hours at 37°C after which the diameters of zone of inhibition were measured and interpreted according to CLSI [42] standard reference values. S. aureus ATCC 29213 was used as quality control strain. Multidrug-resistant (MDR) isolates defined as isolates resistant to three or more antibiotics were also determined.

### 2.8. Data Analysis

Statistical Package for the Social Sciences (SPSS) (version 20) was used to analyze data. The chi-square (*χ*^2^) test was used to analyze the distribution of the organism and enterotoxin genes. Differences were considered significant at *P* ≤ 0.05.

## 3. Results

### 3.1. Distribution of Samples

A total of 289 samples (39, 13.5% milk; 250, 86.5% beef) were collected. Out of 39 milk samples, 17 (43.6%) were from farm S while 22 (56.4%) were collected from farm T. One hundred (40%) beef samples were collected from abattoir A and 150 (60%) from abattoir B.

### 3.2. Contamination of Samples with *S. aureus*

Thirty-two (32) samples (11.1%) were contaminated with *S. aureus.* Of these, 13 (40.6%) were from beef and 19 (59.4%) from milk. *S. aureus* contamination in milk (19/39, 48.7%) was significantly higher than in beef (13/250, 5.2%) (*P* < 0.001) With respect to sample source, milk samples from farm S (9/17, 52.9%) were significantly more contaminated than those from farm T (10/22, 45.5%), while beef samples from abattoir B (10/150, 6.7%) were significantly more contaminated than those from abattoir A (3/100, 3%) (*P* < 0.001) A total of 98 presumptive isolates were obtained from the 32 positive samples based on morphological characteristics. However, only 50 (51.02%) of the isolates tested positive for the *nuc* gene and these were confirmed as *S. aureus.*

### 3.3. Detection of Staphylococcal Enterotoxin Genes

Results on the detection of the enterotoxin genes are recorded in Table 2. All the 50 isolates (100%) confirmed to be *S. aureus* harboured at least one classical enterotoxin gene. The *seb* gene was not detected in any of the isolates. Meanwhile, the *sea* gene (45, 90%) was the most frequently detected enterotoxin gene followed by *see* (38, 76%), *sec* (26, 52%), and *sed* (4, 8%). There was no significant difference (*P* > 0.05) in the distribution of enterotoxin genes in isolates from milk and beef. With the exception of *sea*, all other enterotoxin genes were more frequently observed in isolates from milk than from beef, but the differences were not statistically significant. Generally, all 4 SE genes were detected in isolates from all study sites except *sed*, which was not detected in isolates from abattoir A.

A total of 10 genotypes were observed. A larger proportion of isolates (43/50, 86%) were observed to harbour more than one enterotoxin gene (Table 3). Half of the isolates (50%) carried 2 genes. Isolates with genotype *sea/see* recorded highest prevalence (36%) followed by genotype *sea/sec/see* (34%). However, one isolate (2%) contained four enterotoxin genes: *sea/sec/sed/see*.

### 3.4. Antimicrobial Resistance of *S. aureus* Isolates

**Figure 2:**
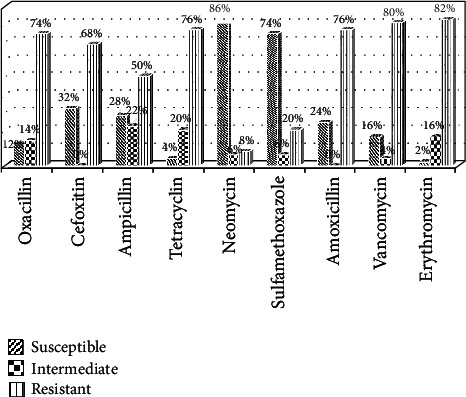
Overall resistance of *S. aureus* isolates to antibiotics.

All the isolates were resistant to at least one of the nine antibiotics tested. Some isolates were intermediate, and others were susceptible at varying rates to these antibiotics. The highest resistance was observed against erythromycin (82%). This was followed by vancomycin (80%), amoxicillin (76%), and tetracycline (76%), while the lowest resistance was against neomycin (8%) (Figure 2).

Generally, higher resistance rates were detected in isolates from milk compared to beef (Table 4). The difference was significant (*P* ≤ 0.05) with respect to amoxicillin, vancomycin, and erythromycin. It was also interesting to note that all samples from farm S were resistant to vancomycin and erythromycin and none was resistant to neomycin. None of the isolates from abattoir B was resistant to trimethoprim-sulfamethoxazole and neomycin (Table 5) indicating that resistance to neomycin was only among isolates from farm T and abattoir A.

Forty-nine (49/50, 98%) isolates were resistant to more than one antibiotic. Isolates exhibited diverse antimicrobial resistance profiles. Multidrug resistance (92%, 46/50) was a common phenomenon. Ten isolates (20%) were resistant to 7 and 5 drugs, respectively, 8 isolates (16%) resistant to 6 drugs, 7 isolates (14%) resistant to 3 drugs, and 5 isolates (10%) each were resistant to 4 drugs and 8 drugs. Twenty-eight antibiotypes were detected (Table 6). One isolate (2%) exhibited resistance to all the nine antibiotics tested. The most predominant antibiotype was antibiotype XXV—OX^R^ FOX^R^ AM^R^ TE^R^ AML^R^ VA^R^ E^R^ (12%, 6/50) followed by antibiotype XXVI—OX^R^ FOX^R^ AM^R^ TE^R^ SXT^R^ AML^R^ VA^R^ E^R^ (8%, 4/50).

## 4. Discussion

*S. aureus* is a commensal that inhabits the skin and mucous membranes of humans and animals. Pathogenic strains are often coagulase positive and have been reported to cause a wide range of diseases in animals and humans all over the world including staphylococcal food poisoning (SFP) [43, 44]. Staphylococcal foodborne disease is one of the most common foodborne diseases worldwide. Food is regarded as a significant vehicle for spreading enterotoxigenic- and antibiotic-resistant strains of this organism [14]. *S. aureus* carrying virulence and antibiotic resistance genes on mobile genetic elements such as plasmids, prophages, and staphylococcal pathogenic islands (SaPIs) can horizontally transfer these determinants between strains resulting in pathogen evolution [45]. Although there is no report of an outbreak of SFP in Cameroon, *S. aureus*, especially MRSA, VISA, and GISA, have been shown to be a safety concern in foods of animal origin [35, 46], underscoring the need for investigating the public health implications of contamination of beef and milk, a very important component of the Cameroonian diet [47]. Studying the occurrence of *S. aureus* in food (at the basic level such as dairy farms and abattoirs) as well as their enterotoxin-producing potential and antibiotic resistance patterns is essential in planning control measures.

In our study, the overall prevalence of *S. aureus* in milk and beef was 11.1%. Apart from cattle with clinical and subclinical infections, other possible sources of contamination include personnel at abattoirs and farms, and the food environment: air, contaminated utensils, soil, and water [48]. We investigated and found contamination in milk and beef from animals which were apparently healthy. Although these animals showed no signs of illness at the time of the study, it is possible that contamination of the samples especially milk with *S. aureus* could be from the skin since these bacteria are normally skin commensals of both humans and animals. Also, since the milk samples were collected manually, the possibility of contamination with livestock associated *S. aureas* cannot be overruled. Consequently, findings of our study could highlight the need for strict implementation of hygiene procedures and quality control of food of animal origin to reduce the risk of health hazards. Our percentage contamination of samples was lower than 37.4% reported in Nairobi, Kenya [49] and 35% in China [50]. A higher rate of contamination of milk (75%) than observed in our study was reported in South Africa by Akindolire et al. [25]. The considerable differences between results of our study and studies elsewhere may be due to differences in geographic area as well as study setting, sampling procedures, sampling sites, sampling time, and sampling at different slaughter and milking process stages.

A significantly higher contamination in milk than beef may be due to the high nutrient content and nearly neutral pH of milk that favours easy and rapid growth of the bacteria, compared to beef. The application of improper milking procedures as reported in other parts of the country [51] could be another contributory factor. Findings of our study support previous reports from Egypt [27] in which contamination of the raw milk (58%) was higher than the raw meat (18%). However, contrary to our study, Abogile and Green [28] in South Africa and Mathenge et al. [49] in Nairobi, Kenya, reported a higher contamination of beef with *S. aureus* than milk. The amount of *S. aureus* in foods depends on factors such as the number of contaminated individuals involved in food handling, poor hygiene practices at the production site, and the rate of animal contamination [52, 53].

SEs are the major virulence factors causing diarrhea and vomiting. The prevalence of SE genes in present study (100%) was higher than 68.2% reported in Portugal [54], 46.24% in Chengdu Province, China [26], 67.8% in Japan [55], and 76.9% in Nairobi, Kenya [49]. These differences could be due to differences in the origin of the milk and beef contaminant which varies from animals, humans, and foods to the environment [45]. Occurrence of SE genes was in this order: *sea* > *see* > *sec* > *sed*; *seb* was not detected. Contrary to our study, Ma et al. [26] reported *seb* as the most common gene. Outbreak investigations of SFP have established SEA as the top major contributor [7, 56]. Mathenge et al. [49] and Rasoul et al. [45] reported a similar trend in the occurrence of these genes. Other studies have reported *sea* as the predominant classical SE gene and variation in the trend of occurrence of other SE genes. Kerouanton et al. [57] reported the pattern: *sea* > *sed* > *seb* > *sec*; *see* was not detected in their study. The prevalence of these genes in our isolates is higher than reported in Iran [45] and in Kenya [49]. Patterns of SE genes might equally vary between different geographical origin and time which can be explained by adaptation of *S. aureus* to different conditions. PCR can only detect the presence of enterotoxin genes but does not prove toxin production. There is therefore need for further studies to investigate SE protein production by these isolates.

Ten genotypes were observed with one isolate harbouring all 4 genes, similar to Mathenge et al. [49]. Detection of isolates from raw beef and raw milk with such diversity in SE genotype is a cause for concern as SFP could be caused by multiple enterotoxins. Previous studies have demonstrated the coexistence of classical SE genes with staphylococcal enterotoxin-like genes [26]. This means that our isolates may also harbour these genes as well. Our study did not investigate new enterotoxin genes and enterotoxin-like genes which could have increased the prevalence of enterotoxigenic *S. aureus* and genotype diversity.

Antibiotic resistance can be transferred to humans via the food chain by consumption of antimicrobial remnants or contaminating resistant bacteria in animal products [58]. In the present study, the high resistance observed to commonly used antibiotics (erythromycin (82%), vancomycin (80%), amoxicillin (76%), tetracycline (70%), oxacillin (74%), and cefoxitin (68%)) is a cause for concern. Findings suggest that these antibiotics could gradually be running out of therapeutic use in study area for treatment of *S. aureus* infections. The detection of a high prevalence of oxacillin resistance (74%) indicates a high prevalence of MRSA among our isolates. We did not investigate the *mecA* and *mecC* gene to confirm this. The indiscriminate use of these antimicrobial agents in animal husbandry could justify the high rates of antimicrobial resistance among isolates [22, 42, 59]. Resistance rates in our study are lower than reported in Nigeria [9], but by far higher than reported in Australia [60] and China [26, 50], reflecting differences in exposure.

High resistance to oxacillin and cefoxitin strongly suggests the possible presence of *mecA* gene (that codes for penicillin-binding protein 2a which causes methicillin resistance) in these isolates. Consequently, further studies to detect the occurrence and dissemination of MRSA in study area are recommended. Statistical analysis comparing antibiotic resistance patterns between milk and beef isolates showed that milk isolates were significantly more resistant to erythromycin, vancomycin, and amoxicillin than isolates from beef. These differences signify differences in antibiotic use in treatment of bacterial-related diseases of animal in dairy farms. Resistant isolates could have originated from colonized/infected milk collectors during milking. The uncontrolled availability of these antibiotics at relatively affordable prices, over the years in Cameroon, has contributed to their misuse and may account for the high resistance rates detected in this study. This constitutes a public health problem because milk and beef consumers are exposed to drug resistant *S. aureus.*

All the 50 isolates were resistant to at least one antibiotic, resulting 28 antibiotypes. Of these 50 isolates, 46 (92%) were multidrug resistant with one isolate showing resistance to all 9 antibiotics tested, suggesting that the transmission of resistance (*R* factor), a plasmid-mediated genetic determinant, may be credited with the development of multidrug resistance (MDR) observed among these isolates. This is not surprising as *S. aureus* is renowned for its ability to develop resistance to multiple antibiotics. Of great concern is the possibility of the spread of resistance to pathogenic and commensal bacteria in the gut flora [61] through horizontal gene transfer mechanisms following the consumption of these contaminated foods. Higher multidrug resistance rate (100%) than detected in our study has been reported in South Africa [62]. *S. aureus* isolates with multiple antibiotic resistance attributes have a negative impact on the treatment of staphylococcal infections, especially in elderly, children, and immune-compromised individuals [63]. However, unlike food infections that rely on antibiotic treatment, SFP is caused by SEs and can progress in the absence of bacteria. However, gut colonization by enterotoxigenic MDR *S. aureus* may get into the blood stream and cause invasive staphylococcal infections (ISI) that affect organs (lung, heart, bone, and kidney) and cause other serious infections such as bacteraemia and meningitis [64].

### 4.1. Limitations of Study

The sample size for this study was estimated to be approximately 340 based on previous reports. However, due to the sociopolitical crisis in Cameroon which posed a great risk to the security of researchers during the period of study, we were not able to collect all the samples as we could not go to other cattle rearing areas. We therefore ended up with a sample size of 289 (250 beef and 39 milk samples). This discrepancy in sample collection may influence results of our study. It is possible that some of the samples might have been contaminated with low numbers of the bacteria which could not be detected by direct plating. The fact that our samples were not enriched prior to culture could lead to false-negative results and this might be a possible explanation for the low prevalence of 11.1%. This study determined the toxin production potential of isolates and did not further investigate the isolates for toxin production. Antibiotic susceptibility of isolates was determined by the disk diffusion technique. MICs were not investigated making it difficult to differentiate between strains that were very sensitive and those with higher MIC. This is important because individuals infected by strains with higher MIC do not respond like those with very sensitive strains. In addition, phenotypic resistance was not confirmed by genotypic methods, constituting another limitation to our study.

## 5. Conclusion

Milk and beef samples in the study area were contaminated with MDR enterotoxigenic strains of *S. aureus* and thus may constitute a potential hazard to consumers. These foods also hold a risk of introduction of resistant microbes in the food chain in the study area. Since food safety is critical to improve health and attain some of the Sustainable Development Goals (SDGs), our findings demand the implementation of high standards of hygiene during the milking process and in the abattoir, and surveillance for S*. aureus* and SEs in foods of animal origin in study area.

## Figures and Tables

**Table 1:** Oligonucleotide primers used for molecular identification of *S. aureus* and detection of enterotoxin genes.

Primer	Sequence	Target gene	Amplicon size (bp)	Reference
Nuc F	GCGATTGATGGTGGATACGGT	*Nuc*	279	[23]
Nuc R	AGCCAAGCCTTGACGAACTAAAGC			
Sea F	GGTTATCAATGTGCGGGTGG	*Sea*	102	[23]
Sea R	CGGCACTTTTTTCTCTTCGG			
Seb F	GTATGGTGGTGTAACTGAGC	*Seb*	164	[23]
Seb R	CCAAATAGTGACGAGTTAGG			
Sec F	AGATGAAGTAGTTGATGTGTATGG	*Sec*	451	[23]
Sec R	CACACTTTTAGAATCAACCG			
Sed F	CCAATAATAGGAGAAAATAAAAG	*Sed*	278	[23]
Sed R	ATTGGTATTTTTTTTCGTTC			
See F	AGGTTTTTTCACAGGTCATCC	*See*	209	[23]
See R	CTTTTTTTTCTTCGGTCAATC			

**Table 2:** Distribution of *S. aureus* enterotoxin genes with respect to sample type.

Gene	Milk isolates (*N* = 35) *n* (%)	Meat isolates (*N* = 15) *n* (%)	Total (*N* = 50) *n* (%)	*P* values
*Sea*	31 (88.6)	14 (93.3)	45 (90)	0.607
*Seb*	0 (0)	0 (0)	0 (0)	
*Sec*	19 (54.3)	7 (46.7)	26 (52)	0.621
*Sed*	3 (8.6)	1 (6.7)	4 (8)	0.820
*See*	27 (77.1)	11 (73.3)	38 (76)	0.773

*N*: number of isolates from type of sample; *n*: number of isolates positive for gene.

**Table 3:** Enterotoxin genotype among the isolates.

Genotype	Source of isolate (number of samples)	Number of isolates with genotype (%)	Number with single, dual, triple, and quadruple genotype (%)
*Sea*	Milk (1), beef (2)	3 (6%)	
*Sec*	Milk (2)	2 (4%)	7 (14%)
*Sed*	Milk (1)	1 (2%)	
*See*	Milk (1)	1 (2%)	
*Sec/see*	Beef (1)	1 (2%)	
*Sea/sec*	Milk (3), beef (2)	5 (10%)	
*Sea/see*	Milk (12), beef (7)	19 (38)	25 (50%)
*Sea/sec/sed*	Milk (1), milk (1)	1 (2%)	17 (34%)
*Sea/sec/see*	Milk (13), beef (3)	16 (32%)	
*Sea/sec/sed/see*	Beef (1)	1 (2%)	1 (2%)
Total		50 (100)	50 (100)

**Table 4:** Distribution of antimicrobial-resistant *S. aureus* isolates with respect to sample type.

Antibiotic	Milk isolates (*N* = 35) *n* (%)	Meat isolates (*N* = 15) *n* (%)	*P* values
Oxacillin	26 (74.3%)	11 (73.3)	0.944
Cefoxitin	26 (74.3%)	8 (53.3%)	0.146
Ampicillin	20 (57.2%)	5 (33.3%)	0.123
Tetracycline	27 (77.1%)	11 (73.3%)	0.773
Neomycin	3 (8.6%)	1 (6.7%)	0.820
Sulfamethoxazole-trimethoprim	9 (25.7%)	1 (6.7%)	0.123
Amoxicillin	31 (88.6%)	7 (46.7%)	0.001
Vancomycin	33 (94.3%)	7 (46.7%)	≤0.001
Erythromycin	33 (94.3%)	8 (53.3)	0.001

*N*: number of isolates tested; *n*: number of isolates positive.

**Table 5:** Antimicrobial resistance of *S. aureus* based on sample source.

Antibiotic	Farm T (*N* = 20)	Farm S (*N* = 15)	Abattoir A (*N* = 5)	Abattoir B (*N* = 10)	*P* values
Ox	15 (75%)	11 (73%)	5 (100%)	6 (60%)	0.425
FOX	13 (65%)	13 (86.7%)	2 (40%)	6 (60%)	0.205
AM	10 (50%)	10 (66.6%)	1 (20%)	4 (40%)	0.276
TE	13 (65%)	14 (93.3%)	3 (60%)	8 (80%)	0.205
N	3 (15%)	0 (0%)	1 (20%)	0 (0%)	0.214
SXT	8 (40%)	1 (6.7%)	1 (20%)	0 (0%)	0.270
AML	18 (90%)	13 (86.7%)	3 (60%)	4 (40%)	0.012
VA	18 (90%)	15 (100%)	3 (60%)	4 (40%)	0.001
E	18 (90%)	15 (100%)	3 (60%)	5 (50%)	0.005

TE: tetracycline; N: neomycin; SXT: sulfamethoxazole; OX: oxacillin; FOX: cefoxitin; AM: ampicillin; AML: amoxicillin; VA: vancomycin; E: erythromycin.

**Table 6:** Resistance patterns of *S. aureus* isolates.

Antibiotype	Resistance pattern	Sample type	No. of isolates with resistance pattern (%)	Frequency of multidrug-resistant isolates
I	TE^R^	Beef	1 (2%)	
II	TE^R^ E^R^	Beef	1 (2%)	
III	OX^R^ AML^R^	Beef	2 (4%)	
IV	OX^R^ VA^R^ E^R^	Beef	1 (2%)	46 (92%)
V	OX^R^ FOX^R^ TE^R^	Beef	1 (2%)	
VI	AML^R^ VA^R^ E^R^	Milk	3 (6%)	
VII	FOX^R^ TE^R^ E^R^	Beef	2 (4%)	
VIII	FOX^R^ AM^R^ TE^R^ VA^R^	Milk	1 (2%)	
IX	FOX^R^ TE^R^ VA^R^ E^R^	Milk	1 (2%)	
X	OX^R^ FOX^R^ AM^R^TE^R^	Beef	1 (2%)	
XI	OX^R^ FOX^R^TE^R^ E^R^	Beef	1 (2%)	
XII	OX^R^ AM^R^ AML^R^ VA^R^	Beef	1 (2%)	
XIII	OX^R^ TE^R^ AML^R^ VA^R^ E^R^	Milk	3 (6%)	
XIV	OX^R^ FOX^R^ TE^R^ AML^R^ VA^R^	Beef	1 (2%)	
XV	OX^R^ FOX^R^ AM^R^ TE^R^ VA^R^	Milk	2 (4%)	
XVI	OX^R^ AM^R^ TE^R^ AML^R^ VA^R^	Beef	1 (2%)	
XVII	OX^R^ FOX^R^TE^R^ VA^R^ E^R^	Milk	2 (4%)	
XVIII	OX^R^ SXT^R^ AML^R^ VA^R^ E^R^	Milk	1 (2%)	
XIX	OX^R^ FOX^R^ TE^R^ AML^R^VA^R^ E^R^	Milk	3 (6%)	
XX	FOX^R^ AM^R^ TE^R^ AML^R^ VA^R^ E^R^	Milk	3 (6%)	
XXI	FOX^R^ AM^R^ SXT^R^ AML^R^ VA^R^ E^R^	Milk	1 (2%)	
XXII	OX^R^ FOX^R^ AM^R^ AML^R^ VA^R^ E^R^	Beef	1 (2%)	
XXIII	OX^R^ AM^R^ TE^R^ SXT^R^ AML^R^ VA^R^ E^R^	Beef	2 (4%)	
XXIV	OX^R^FOX^R^ TE^R^ N^R^ AML^R^ VA^R^ E^R^	Beef, milk	2 (4%)	
XXV	OX^R^ FOX^R^ AM^R^ TE^R^ AML^R^ VA^R^ E^R^	Milk	6 (12%)	
XXVI	OX^R^ FOX^R^ AM^R^ TE^R^ SXT^R^ AML^R^ VA^R^ E^R^	Milk	4 (8%)	
XXVII	OX^R^ FOX^R^ AM^R^ N^R^ SXT^R^ AML^R^ VA^R^ E^R^	Milk	1 (2%)	
Total			50 (100%)	46 (92%)

## Data Availability

Data used to support the findings of this study are included within the article.

## Abbreviations

CLSI: Clinical and Laboratory Standards Institute
ISI: Invasive staphylococcal infections
MDR: Multidrug resistance
MRSA: Methicillin-resistant *Staphylococcus aureus*
PBP2a: Penicillin-binding protein 2a
SaPIs: Staphylococcal pathogenicity islands
SCCmec: Staphylococcal chromosome cassette mec
SDGs: Sustainable Development Goals
SEs: Staphylococcal enterotoxins
SEA-V: Staphylococcal enterotoxin A-V
SFP: Staphylococcal food poisoning.
